# Synthesis and *In vitro* cytotoxic activity evaluation of (*E*)-16-(substituted benzylidene) derivatives of dehydroepiandrosterone

**DOI:** 10.1186/2008-2231-21-34

**Published:** 2013-05-01

**Authors:** Mohsen Vosooghi, Hoda Yahyavi, Kouros Divsalar, Hashem Shamsa, Asma Kheirollahi, Maliheh Safavi, Sussan Kabudanian Ardestani, Sareh Sadeghi-Neshat, Negar Mohammadhosseini, Najmeh Edraki, Mehdi Khoshneviszadeh, Abbas Shafiee, Alireza Foroumadi

**Affiliations:** 1 Department of Medicinal Chemistry, Faculty of Pharmacy & Pharmaceutical Sciences Research Center, Tehran University of Medical Sciences, Tehran, Iran; 2 Neuroscience Research Center, Kerman University of Medical Sciences, Kerman, Iran; 3 Institute of Biochemistry and Biophysics, Department of Biochemistry, University of Tehran, P.O. Box 13145–1384, Tehran, Iran; 4 Medicinal & Natural Products Chemistry Research Center, Shiraz University of Medical Sciences, Shiraz, Iran

**Keywords:** Synthesis, Dehydroepiandrosterone (DHEA), MTT assay, Cytotoxic activity

## Abstract

**Background and the purpose of the study:**

Modified androsterone derivatives are class of steroidal compounds with potential anticancer properties. Various steroidal derivatives containing substitution at position 16 have shown diversified pharmacological activities. In the present study, a new series of cytotoxic 16-(substituted benzylidene) derivatives of dehydroepiandrosterone (DHEA) were synthesized and evaluated against three different cancer cell lines.

**Methods:**

The cytotoxic 16-(substituted benzylidene) derivatives of DHEA were synthesized via aldol condensation of DHEA with corresponding benzaldehyde derivatives. The cytotoxic activity of synthesized derivatives was evaluated against three different cancer cells including KB, T47D and SK-N-MC cell lines by MTT reduction colorimetric assay.

**Results:**

The results indicated that 16-(substituted benzylidene) derivatives of DHEA could be served as a potent anti-cancer agent. The 3-cholro benzylidene derivatives of DHEA was the most potent synthesized derivative especially against KB and T47D cell lines (IC_50_ values were 0.6 and 1.7 μM; respectively).

**Conclusion:**

The cytotoxic potential of novel benzylidene derivatives of DHEA is mainly attributed to the position and nature of the substituted group on the benzylidene pendant.

## Introduction

Steroidal derivatives are important class of synthetic and naturally occurring compounds, which have exhibited different biological properties [1-3] and attracted profound attention for development of potent pharmacological agents for treatments of various diseases [4] including: cardiovascular disease [5], adrenal insufficiencies [6], autoimmune disorders [7], fungal and microbial infections [8,9]. Furthermore, different steroidal derivatives have been considered as potent anti-cancer agents for the treatment of leukemia [4], breast cancer [10-12], prostate cancer [13] and brain tumors [14].

**Figure 1:**
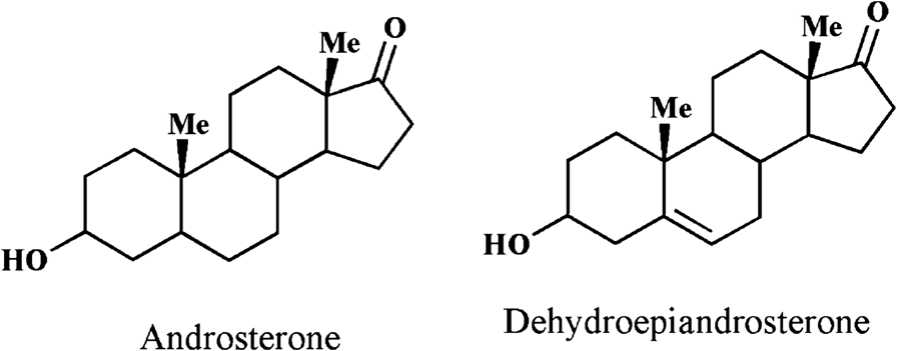
Chemical structures of androsterone and dehydroepiandrosterone.

Several natural and modified steroidal derivatives have been previously described in the literatures as potent cytotoxic agents [15-17]. In this regard, different derivatives of androsterone (3α-hydroxy-5α-androstan-17-one) have been excessively studied as potent anti-cancer agents (Figure 1) [18,19]. Recently, the significant cytotoxic and aromatase inhibitory potential of a large number of androsterone derivatives containing substitution at position 16 have been reported [12,20,21]. Bansal et. al demonstrated the effectiveness of different 16E-arylidenosteroids as potential anticancer and anti-aromatase scaffold against estrogen-dependent breast cancer and different human tumor cell lines [12,20]. The cytotoxic mechanistic study of α,β-unsaturated carbonyl derivatives revealed that compounds containing described functional group can cause alteration and mis-folding of proteins through the formation of adducts with reactive thiol groups of proteins [22]. For this reason, the α-β unsaturated androstrone derivatives containing exocyclic double bond at C16 position could be served as potent chemotherapeutic agents. Dehydroepiandrosterone (DHEA), also known as androstenolone (3β-hydroxyandrost-5-en-17-one) is an androsterone derivative and important endogenous steroid hormone which plays an important role as intermediate for biosynthesis of androgens and estrogen hormones [23]. Apart from its different biological potential, DHEA demonstrated antiproliferative and antiapoptotic effects on different cancer cell lines [24-26].

In the course of our ongoing study for the synthesis and biological evaluation of potential anticancer agents [27-34], herein, we investigate the synthesis and cytotoxic activity evaluation of a new series of 16-(substituted benzylidene) derivatives of DHEA taking into account the structural necessities for cytotoxic activity of these derivatives. The aim of this study was to investigate the structural requirements affecting the cytotoxic potential of modified steroidal compounds.

## Material and methods

### Chemistry

All starting materials, reagents, and solvents were prepared from Merck AG (Germany). Thin layer chromatography (TLC) using various solvents of different polarities was applied for determination of the purity of the synthesized compounds. Melting points were determined on a Kofler hot stage apparatus (Vienna, Austria) and are uncorrected. ^1^H-NMR spectra were recorded using a Bruker 400 spectrometer (Bruker, Rheinstatten, Germany), and chemical shifts are expressed as δ (ppm) with tetramethylsilane (TMS) as internal standard. The IR spectra were recorded using a Shimadzu 470 (Shimadzu, Tokyo, Japan) spectrophotometer (potassium bromide disks).The mass spectra were recorded on a Finnigan TSQ-70 spectrometer (Finnigan, USA) at 70 eV.

#### General procedure for the preparation of the (E)-16-(substituted benzylidene) dehydroepiandrosterone derivatives 1a-m using aldol Condensation

The appropriate aldehyde was added to a mixture of DHEA (1.0 g, 3.47 mmol) and NaOH (1.75 g) in methanol (20 ml). The reaction mixture was stirred for 1 h at room temperature. The completion of reaction was confirmed using analytical thin layer chromatography. After completion, the reaction mixture was poured into ice-water. The final precipitate was filtered; washed with cold water, dried under reduced pressure and crystallized in methanol.

#### (E)-16-(2-Chlorobenzylidene)-1,3,4,7,8,9,10,11,12,13,15,16-dodecahydro-3-hydroxy-10,13-dimethyl-2H-cyclopenta[a]phenanthren-17(14H)-one (1a)

Yield: 23%; mp=213-214◦°C; IR (KBr, ν_max_, cm^-1^): 3475(OH), 1725(C=O).^1^HNMR (400 MHz, CDCl_3_): 1.00(s, 3H, CH_3_), 1.07 (s, 3H, CH_3_), 3.52-3.63(m, 1H, CH-OH), 5.40(s, 1H, H_vinyl_), 7.28-7.32(m, 2H, H_phenyl_), 7.42-7.46(m, 1H, H_phenyl_), 7.52-7.56(m,1H, H_phenyl_). MS (EI) *m/z* (%): 412 (M^+^+2, 31), 410(M^+^, 100).

#### (E)-16-(3-Chlorobenzylidene)-1,3,4,7,8,9,10,11,12,13,15,16-dodecahydro-3-hydroxy-10,13-dimethyl-2H-cyclopenta[a]phenanthren-17(14H)-one (1b)

Yield: 30%; mp= 199-202◦°C; IR (KBr, ν_max_, cm^-1^): 3219 (OH), 1708 (C=O).^1^HNMR (400 MHz, CDCl_3_): 0.99(s, 3H, CH_3_), 1.07(s, 3H, CH_3_), 3.48-3.60(m, 1H, CH-OH), 5.41(s, 1H, H_vinyl_), 7.33-7.43(m, 4H, H_phenyl_). MS (EI) *m/z* (%): 412 (M^+^+2, 5), 410 (M^+^, 15).

#### (E)-16-(4-Chlorobenzylidene)-1,3,4,7,8,9,10,11,12,13,15,16-dodecahydro-3-hydroxy-10,13-dimethyl-2H-cyclopenta[a]phenanthren-17(14H)-one (1c)

Yield: 27%; mp=229-231◦°C; IR (KBr, ν_max_, cm^-1^): 3416(OH), 1710(C=O). ^1^HNMR (400 MHz, CDCl_3_): 0.98(s, 3H, CH_3_), 1.07(s, 3H, CH_3_), 3.53-3.54(m, 1H, CH-OH), 5.40(s, 1H, Hvinyl), 7.39(dd, 1H, H_phenyl_, *J*= 8.5Hz ),7.46(dd, 1H, H_phenyl_, *J*= 8.5Hz ). MS (EI) *m/z* (%): 412 (M^+^+2, 10), 410 (M^+^, 28), 378(18), 351(4), 300(19), 268(10), 214(22), 150(100), 91(87), 79(100).

#### (E)-16-(2,4-Dichlorobenzylidene)-1,3,4,7,8,9,10,11,12,13,15,16-dodecahydro-3-hydroxy-10,13-dimethyl-2H-cyclopenta[a]phenanthren-17(14H)-one (1d)

Yield: 35%; mp=203-205◦°C; IR (KBr, ν_max_, cm^-1^): 3472 (OH), 2929(CH aliphatic), 1710(C=O).^1^H-NMR (DMSO-*d*_6_): ^1^HNMR (400 MHz, CDCl_3_): 1.03(s, 3H, CH_3_), 1.11(s, 3H, CH_3_), 3.55-3.56(m, 1H, CH-OH), 5.40(s, 1H, H_vinyl_), 7.48-7.49(m, 2H, H_phenyl_), 7.48(s, 1H, H_phenyl_), 7.70-7.72(m, 1H, H_phenyl_). MS (EI) *m/z* (%): 445(M^+^,10), 410(100), 343(18), 297(10), 213(11), 186(18), 105(9), 57(18).

#### (E)-16-(4-Fluorobenzylidene)-1,3,4,7,8,9,10,11,12,13,15,16-dodecahydro-3-hydroxy-10,13-dimethyl-2H-cyclopenta[a]phenanthren-17(14H)-one (1e)

Yield: 46%; mp=234-236◦°C; IR (KBr, ν_max_, cm^-1^): 3426(OH), 1716(C=O). ^1^HNMR (400 MHz, CDCl_3_): 0.98(s, 3H, CH_3_), 1.07(s, 3H, CH_3_), 3.48-3.58(m, 1H, CH-OH), 5.40(s, 1H, H_vinyl_), 7.10(t, 1H, H_phenyl_, *J*= 8.5 Hz), 7.52(t, 1H, H_phenyl_, *J*= 8.5Hz). MS (EI) *m/z* (%): 395(M^+^, 38), 377(10), 284(10), 232(32), 203(39), 145(25), 134(100), 109(25), 82(12).

#### (E)-16-(3-Bromobenzylidene)-1,3,4,7,8,9,10,11,12,13,15,16-dodecahydro-3-hydroxy-10,13-dimethyl-2H-cyclopenta[a]phenanthren-17(14H)-one (1f)

Yield: 28%; mp=222-234◦°C, IR (KBr, ν_max_, cm^-1^): 3471(OH), 1709 (C=O). ^1^HNMR (400 MHz, CDCl_3_):0.95(s, 3H, CH_3_), 1.04(s, 3H, CH_3_), 3.48-3.60( m, 1H, CH-OH), 5.40(s, 1H, H_Vinyl_), 7.29(t, 1H, H_phenyl_, *J*= 6.4Hz), 7.35(s, 1H, H_phenyl_), 7.46(d, 1H, H_phenyl_, *J*= 6.4Hz), 7.49(d, 1H, H_phenyl_, *J*= 6.4Hz). MS (EI) *m/z* (%): 456(M^+^, 26), 454(M^+^, 26), 436(46), 424(32), 343(26), 315(18), 263(32), 213(38).

#### (E)-16-(5-Bromo-2-hydroxybenzylidene)-1,3,4,7,8,9,10,11,12,13,15,16-dodecahydro-3-hydroxy-10,13-dimethyl-2H-cyclopenta[a]phenanthren-17(14H)-one (1g)

Yield: 46%; mp=208-210◦°C; IR (KBr, ν_max_, cm^-1^): 3464(OH), 1709(C=O). ^1^HNMR (400 MHz, CDCl_3_): 1.00(s, 3H, CH_3_), 1.07 (s, 3H, CH_3_), 3.42-3.60(m, 1H, CH-OH),5.40(s, 1H, H_vinyl_), 6.83(dd, 1H, H_phenyl_, *J*= 8.4Hz), 7.32(dd, 1H, H_phenyl_, *J*= 8.4Hz), 7.52(d, 1H, H_phenyl_, *J*= 8.4Hz). MS (EI) *m/z* (%): 472 (M^+^+2, 95), 470(M^+^, 95).

#### (E)-16-(2-(Trifluoromethyl)benzylidene)-1,3,4,7,8,9,10,11,12,13,15,16-dodecahydro-3-hydroxy-10,13-dimethyl-2H-cyclopenta[a]phenanthren-17(14H)-one (1h)

Yield: 34%; mp = 208-210◦°C; IR (KBr, ν_max_, cm^-1^): 3456(OH), 1724(C=O).^1^HNMR (400 MHz, CDCl_3_): 1.00(s, 3H, CH_3_), 1.06 (s, 3H, CH_3_), 3.48-3.60(m, 1H, CH-OH), 5.38(s, 1H, H_vinyl_), 7.42-48(m, 1H, H_phenyl_) 7.56-7.60 (m, 2H, H_phenyl_), 7.70-7.75(m, 2H, H_phenyl_-H_Vinyl_ ). MS (EI) *m/z* (%): 445 (M^+^+1, 14), 444(M^+^, 100).

#### (E)-16-(4-(Trifluoromethyl)benzylidene)-1,3,4,7,8,9,10,11,12,13,15,16-dodecahydro-3-hydroxy-10,13-dimethyl-2H-cyclopenta[a]phenanthren-17(14H)-one (1i)

Yield: 27%; mp=244-246◦°C; IR (KBr, ν_max_, cm^-1^): 3215(OH), 1708(C=O). ^1^HNMR (400 MHz, CDCl_3_): 0.99(s, 3H, CH_3_), 1.08(s, 3H, CH_3_), 3.48-3.60(m, 1H, CH-OH), 7.40(s, 1H, H_vinyl_), 7.38-7.46(m, 2H, H_phenyl_), 7.64(dd, 2H, H_phenyl_, *J*=8.8Hz). MS (EI) *m/z* (%): 445 (M^+^+1, 21), 444(M^+^, 100).

#### (E)-16-(4-Methylbenzylidene)-1,3,4,7,8,9,10,11,12,13,15,16-dodecahydro-3-hydroxy-10,13-dimethyl-2H-cyclopenta[a]phenanthren-17(14H)-one (1j)

Yield: 78%; mp=238-240◦°C; IR (KBr, ν_max_, cm^-1^): 3421(OH), 1716(C=O). ^1^HNMR (400 MHz, CDCl_3_): 0.98(s, 3H, CH_3_), 1.07(s, 3H, CH_3_), 1.57(s, 3H, CH_3_), 3.45-3.50(m, 1H, CH-OH), 5.42(s, 1H, H_vinyl_), 7.25(dd, 1H, H_Phenyl_, *J*=8.4Hz), 7.44(dd, 1H, H_Phenyl_, *J*=8.4 Hz). MS (EI) *m/z* (%): 391 (M^+^+1, 9), 390 (M^+^, 44), 376(4), 131(100).

#### (E)-16-(4-Methoxybenzylidene)-1,3,4,7,8,9,10,11,12,13,15,16-dodecahydro-3-hydroxy-10,13-dimethyl-2H-cyclopenta[a]phenanthren-17(14H)-one(1k)

Yield: 25%; mp =225-227◦°C; IR (KBr, ν_max_, cm^-1^): 3454(OH), 1712(C=O). ^1^HNMR (400 MHz, CDCl_3_): 0.97(s, 3H, CH_3_), 1.07(s, 3H, CH_3_), 3.85(s, 3H, OCH_3_), 3.50-3.60(m, 1H, CH-OH), 5.40(s, 1H, H_vinyl_), 6.94(dd, 1H, H_phenyl_, *J*=8.4Hz), 7.40(s, 1H, H_vinyl_), 7.51(dd, 1H, H_phenyl_, *J*=8.4Hz). MS (EI) *m/z* (%): 407(M^+^+1, 7), 406(M^+^, 29), 408(100).

#### (E)-16-(2,3,4-Trimethoxybenzylidene)-1,3,4,7,8,9,10,11,12,13,15,16-dodecahydro-3-hydroxy-10,13-dimethyl-2H-cyclopenta[a]phenanthren-17(14H)-one (1l)

Yield: 20%; mp=199-201◦°C; IR (KBr, ν_max_, cm^-1^): 3429(OH), 1717(C=O).^1^HNMR (400 MHz, CDCl_3_): 0.98(s, 3H, CH_3_), 1.07(s, 3H, CH_3_), 3.90(s, 9H, 3OCH_3_), 5.39(s, 1H, H_vinyl_), 6.72(d, 1H, H_Phenyl_, *J*=8.4Hz), 7.27(d, 1H, H_Phenyl,_*J*=8.4Hz). MS (EI) *m/z* (%):467(M^+^+1, 28), 466(M^+^, 100).

#### (E)-16-(4-(Dimethylamino)benzylidene)-1,3,4,7,8,9,10,11,12,13,15,16-dodecahydro-3-hydroxy-10,13-dimethyl-2H-cyclopenta[a]phenanthren-17(14H)-one (1m)

Yield: 35%; mp=216-218◦°C; IR (KBr, ν_max_, cm^-1^): 3521(OH), 1721(C=O). ^1^HNMR (400 MHz, CDCl_3_): 0.96(s, 3H, CH_3_), 1.07(s, 3H, CH_3_), 3.03(s, 6H, 2CH_3_), 3.42-3.60(m, 1H, CH-OH), 5.42(s, 1H, H_vinyl_), 6.71(d, 1H, H_phenyl_, *J*= 8.3Hz), 7.47(d, 1H, H_phenyl_, *J*= 8.3Hz). MS (EI) *m/z* (%): 420(M^+^+1, 32), 419(M^+^, 10).

### Biological assay

#### Cell lines and cell culture

The synthesized compounds were tested against three different human cancer cell lines including KB (human nasopharyngeal epidermoid carcinoma), T47D (human breast cancer) and SK-N-MC (human neuroblastoma) cells. The cell lines were purchased from the National Cell Bank of Iran (NCBI). The cells were grown in RPMI- 1640 medium (Gibco BRL) supplemented with 10% heat inactivated fetal calf serum (Gibco BRL), 100 μg/mL streptomycin, and 100 U/mL penicillin, in a humidified air atmosphere at 37°C with 5% CO2.

#### In vitro cytotoxicity assay

The *in vitro* cytotoxic activity of each synthesized derivatives 1a-m was investigated using MTT colorimetric assay [35]. Briefly, each cell line in log-phase of growth was harvested by trypsinization followed by resuspension in complete growth medium to give a total cell count of 5×10^4^ cells/ml. The resulted cell suspension was seeded into the wells of 96-well plates (Nunc, Denmark). The plates were incubated overnight in a humidified air atmosphere at 37°C with 5% CO_2_. After the incubation period, 5 μL of the media containing various concentrations of the compounds was added per well in triplicate followed by further incubation for 24 h. The final maximum concentration of DMSO was 0.1%. Etoposide was used as positive control for cytotoxic activity, while three different wells containing evaluated cancer cells cultured in 200 μL of complete medium were used as negative controls of cell viability. After incubation, the medium was discarded and 200 μl phenol red-free RPMI containing MTT (final concentration 1 mg/ml), was added to each well. The test plate was incubated for 4h. The culture medium was then replaced with 100 μL of DMSO and the absorbance of each well was measured by using a micro plate reader (Gen5, Power wave xs2, BioTek, America) at 492 nm. Each set of experiments was independently performed three times. The concentration causing 50% cell growth inhibition (IC_50_) compared with the control was calculated using concentration-response curves by regression analysis.

## Results and discussion

**Figure 2:**
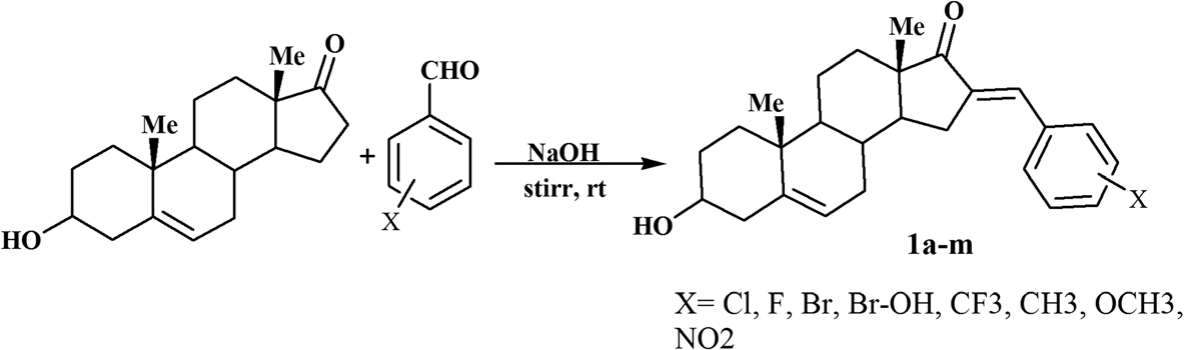
Synthetic protocol for compounds 1a-m.

The benzylidene-substituted DHEA derivatives 1a-m were synthesized through the aldol condensation [36] of DHEA with corresponding benzaldehyde derivatives (Figure 2).

The *in vitro* cytotoxic activity of synthesized compounds 1a-m was investigated against three different cancer cell lines including KB, T47D and SK-N-M cells. The percentage of growth inhibition was assessed using MTT reduction assay versus controls not treated with test compounds. The 50% growth inhibitory concentration (IC_50_) for each compound was determined and presented in Table 1. The data for etoposide was also included.

**Table 1:** Chemical structures and in vitro cytotoxic activity of compounds 1a-m assessed by MTT reduction assay


**IC**_**50 **_**(μ *M *)**^**a**^				
**Compound**	**Ar**	**KB**	**T47D**	**SK-N-MC**
**1a**		2.9(±10.1)	9.6 ±3.1	13.2± 2.2
**1b**		0.6(±2.0)	1.7±14.8	10.0± 3
**1c**		>100	>100	>100
**1d**		>100	>100	3.6±13.26
**1e**		>100	>100	>100
**1f**		6.5(±21.1)	>100	3.6±40.7
**1g**		>100	>100	16.3(±55.9)
**1h**		1.2(±3.3)	3.6(±7.3)	2.7(±3.6)
**1i**		2.5(±8.3)	2.4(±9.6)	2.0(±5.3)
**1j**		1.7(±12.5)	7.6(±17.4)	1.0(±17.4)
**1k**		1.3(±18)	4.1(±12.3)	5.9(±14.1)
**1l**		>100	>100	>100
**1m**		>100	>100	>100
**Etoposide**	-	2.8(±16.8)	1.2(±8)	3.9(±8.3)

^a^ Values in parentheses represent the average of 3–4 experiments ± S.E.M.

The results of cytotoxic data indicate that most of synthesized compounds showed moderate to strong cytotoxic potential in all three cell lines. Based on the cytotoxic data, the following structure-activity relationship may be developed:

Introduction of different substitutes such as chlorine, trifluoromethyl, methoxy and methyl groups into the *ortho* or *meta* position of benzylidene moiety, resulted in enhanced cytotoxic potential of benzylidene derivatives of DHEA.

The compounds containing chlorine, nitro and fluorine substitutes at *para* position of benzylidene pendant, were almost inactive against all three evaluated cell lines (IC_50_>100 μM). Whereas, substitution of methoxy, methyl and trifluoromethyl groups into the *para* position (compounds 1i-j), resulted in enhanced cytotoxic potential of corresponding derivatives, e.g. the corresponding IC_50_ values of *para*-methyl benzylidene derivative 1j in KB, T47D and SK-N-MC cell lines were 1.7, 7.6 and 1.0 μM, respectively.

The 3-chloro benzylidene derivatives of DHEA, compound 1b, was the most potent synthesized derivative especially against KB and T47D cell lines (IC_50_ values were 0.6 and 1.7 μM; respectively) which were comparable with etoposide (IC_50_= 2.8 and 1.2 μM; respectively).

Based on the above finding it might be deduced that 16-(substituted benzylidene) derivatives of DHEA could be served as a potent anti-cancer agents. The cytotoxic potential of described compounds is mainly attributed to the position and nature of the substituted group on the benzylidene pendant. The *ortho* or *meta* positions of the benzylidene group could well accommodate different substitute in order to afford potent cytotoxic derivatives of this types.

## Conclusion

A new series of cytotoxic 16-(substituted benzylidene) derivatives of DHEA were synthesized and evaluated against three different cancer cells including KB, T47D and SK-N-MC cell lines by MTT reduction colorimetric assay. The cytotoxic potential of these novel benzylidene derivatives of DHEA is mainly attributed to the position and nature of the substituted group on the benzylidene pendant.

## Competing interests

The authors declare that they have no competing interests.

## Authors’ contributions

MV: Design and synthesis of target compounds. HY: Synthesis of some intermediates and target compounds, KD: performing the biological tests, HS: Synthesis of some target compounds, MS: performing the cytotoxic test, SKA: Supervision of biological tests, NM: collaboration in identification of synthesized compounds, NE: collaboration in identifying of the structures of target compounds, manuscript preparation, MK: collaboration in manuscript preparation, AS: Collaboration in identifying the structures of target compounds, AF: Design of target compounds and supervision of the synthetic and pharmacological parts. All authors read and approved the final manuscript.

## References

[B1] Mensah-Nyagan AG Meyer L Schaeffer V Kibaly C Patte-Mensah C Evidence for a key role of steroids in the modulation of pain Psychoneuroendocrino 2009 34 169 177 10.1016/j.psyneuen.2009.06.004 19577851

[B2] Ibrahim-Ouali M Recent advances in oxasteroids chemistry Steroids 2007 72 475 508 10.1016/j.steroids.2007.03.004 17499322

[B3] Bhatti HN Khera RA Biological transformations of steroidal compounds: A review Steroids 2012 77 1267 1290 10.1016/j.steroids.2012.07.018 22910289

[B4] Bansal R Guleria S Thota S Bodhankar SL Patwardhan MR Zimmer C Design, synthesis and evaluation of novel 16-imidazolyl substituted steroidal derivatives possessing potent diversified pharmacological properties Steroids 2012 77 621 629 10.1016/j.steroids.2012.02.005 22366075

[B5] Dubey RK Oparil S Imthurn B Jackson EK Sex hormones and hypertension Cardiovasc Res 2002 53 688 708 10.1016/S0008-6363(01)00527-2 11861040

[B6] Holst JP Soldin SJ Tractenberg RE Guo T Kundra P Verbalis JG Use of steroid profiles in determining the cause of adrenal insufficiency Steroids 2007 72 71 84 10.1016/j.steroids.2006.11.001 17157339 PMC1952234

[B7] Auci DL Reading CL Frincke JM 7-Hydroxy androstene steroids and a novel synthetic analogue with reduced side effects as a potential agent to treat autoimmune diseases Autoimmun Rev 2009 8 369 372 10.1016/j.autrev.2008.11.011 19071234

[B8] Jursic BS Upadhyay SK Creech CC Neumann DM Novel and efficient synthesis and antifungal evaluation of 2,3-functionalized cholestane and androstane derivatives Bioorg Med Chem Lett 2010 20 7372 7375 10.1016/j.bmcl.2010.10.044 21036611

[B9] Banday AH Iqbal Zargar M Ganaie BA Synthesis and antimicrobial studies of chalconyl pregnenolones Steroids 2011 76 1358 1362 10.1016/j.steroids.2011.07.001 21771607

[B10] Billich A Nussbaumer P Lehr P Stimulation of MCF-7 breast cancer cell proliferation by estrone sulfate and dehydroepiandrosterone sulfate: inhibition by novel non-steroidal steroid sulfatase inhibitors J Steroid Biochem 2000 73 225 235 10.1016/S0960-0760(00)00077-7 11070351

[B11] Saha P Fortin S Leblanc V Parent S Asselin E Berube G Design, synthesis, cytocidal activity and estrogen receptor alpha affinity of doxorubicin conjugates at 16alpha-position of estrogen for site-specific treatment of estrogen receptor positive breast cancer Steroids 2012 77 1113 1122 10.1016/j.steroids.2012.06.004 22801351

[B12] Bansal R Guleria S Thota S Hartmann RW Zimmer C Synthesis and biological evaluation of 16E-arylidenosteroids as cytotoxic and anti-aromatase agents Chem Pharma Bull 2011 59 327 331 10.1248/cpb.59.327 21372413

[B13] Gauthier S Martel C Labrie F Steroid derivatives as pure antagonists of the androgen receptor J Steroid Biochem 2012 132 93 104 10.1016/j.jsbmb.2012.02.006 22449547

[B14] Sheridan PJ Blum K Trachtenberg MC **Steroid receptors and disease: cancer, autoimmune, bone, and circulatory disorders** *Marcel Dekker Inc* 1988 289 564

[B15] Li C Qiu W Yang Z Luo J Yang F Liu M Stereoselective synthesis of some methyl-substituted steroid hormones and their in vitro cytotoxic activity against human gastric cancer cell line MGC-803 Steroids 2010 75 859 869 10.1016/j.steroids.2010.05.008 20493894

[B16] Li Y Huang J Liu J Yan P Liu H Sun Q Synthesis and cytotoxicity of 17E-(2-aryl-2-oxo-1-ethylidene)-5alpha-androstane derivatives Steroids 2011 76 1615 1620 10.1016/j.steroids.2011.10.003 22027219

[B17] Duh CY Lo IW Wang SK Dai CF New cytotoxic steroids from the soft coral Clavularia viridis Steroids 2007 72 573 579 10.1016/j.steroids.2007.03.010 17485104

[B18] Banday AH Singh S Alam MS Reddy DM Gupta BD Sampath Kumar HM Synthesis of novel steroidal D-ring substituted isoxazoline derivatives of 17-oxoandrostanes Steroids 2008 73 370 374 10.1016/j.steroids.2007.10.014 18166206

[B19] Krstic NM Bjelakovic MS Pavlovic VD Robeyns K Juranic ZD Matic I New androst-4-en-17-spiro-1,3,2-oxathiaphospholanes. Synthesis, assignment of absolute configuration and in vitro cytotoxic and antimicrobial activities Steroids 2012 77 558 565 10.1016/j.steroids.2012.01.021 22342468

[B20] Bansal R Thota S Karkra N Minu M Zimmer C Hartmann RW Synthesis and aromatase inhibitory activity of some new 16E-arylidenosteroids Bioorg Chem 2012 45 36 40 23064126 10.1016/j.bioorg.2012.08.005

[B21] Chattopadhaya R Jindal DP Minu M Gupta R Synthesis and cytotoxic studies of hydroximino derivatives of some 16E-arylidenosteroids Arzneimittel-Forsch 2004 54 551 556 15500202 10.1055/s-0031-1297011

[B22] Bradshaw TD Matthews CS Cookson J Chew EH Shah M Bailey K Elucidation of thioredoxin as a molecular target for antitumor quinols Cancer Res 2005 65 3911 3919 10.1158/0008-5472.CAN-04-4141 15867391

[B23] Mo Q Lu SF Simon NG Dehydroepiandrosterone and its metabolites: differential effects on androgen receptor trafficking and transcriptional activity J Steroid Biochem 2006 99 50 58 10.1016/j.jsbmb.2005.11.011 16524719

[B24] Yang NC Jeng KC Ho WM Hu ML ATP depletion is an important factor in DHEA-induced growth inhibition and apoptosis in BV-2 cells Life Sci 2002 70 1979 1988 10.1016/S0024-3205(01)01542-9 12148690

[B25] Loria RM Immune up-regulation and tumor apoptosis by androstene steroids Steroids 2002 67 953 966 10.1016/S0039-128X(02)00043-0 12398992

[B26] Tworoger SS Sluss P Hankinson SE Association between plasma prolactin concentrations and risk of breast cancer among predominately premenopausal women Cancer Res 2006 66 2476 2482 10.1158/0008-5472.CAN-05-3369 16489055

[B27] Safavi M Esmati N Ardestani SK Emami S Ajdari S Davoodi J Shafiee A Foroumadi A Halogenated flavanones as potential apoptosis-inducing agents: synthesis and biological activity evaluation Eur J Med Chem 2012 58 573 580 23174316 10.1016/j.ejmech.2012.10.043

[B28] Nakhjiri M Safavi M Alipour E Emami S Atash AF Jafari-Zavareh M Asymmetrical 2, 6-bis (benzylidene) cyclohexanones: Synthesis, cytotoxic activity and QSAR study Eur J Med Chem 2012 50 113 123 22341788 10.1016/j.ejmech.2012.01.045

[B29] Aryapour H Mahdavi M Mohebbi SR Zali MR Foroumadi A Anti-proliferative and apoptotic effects of the derivatives from 4-aryl-4H-chromene family on human leukemia K562 cells Arch Pharm Res 2012 35 1573 1582 10.1007/s12272-012-0908-y 23054714

[B30] Rafinejad A Fallah-Tafti A Tiwari R Shirazi AN Mandal D Shafiee A Parang K Foroumadi A Akbarzadeh T 4-Aryl-4*H*-naphthopyrans derivatives: One-pot synthesis, evaluation of Src kinase inhibitory and anti-proliferative activities DARU J Pharmaceut Sci 2012 20 100 10.1186/2008-2231-20-100 PMC3599540 23351304

[B31] Firoozpour L Edraki N Nakhjiri M Emami S Safavi M Ardestani SK Cytotoxic activity evaluation and QSAR study of chromene-based chalcones Arch Pharm Res 2012 35 2117 2125 10.1007/s12272-012-1208-2 23263805

[B32] Bazl R Ganjali MR Saboury AA Foroumadi A Nourozi P Amanlou M A new strategy based on pharmacophore-based virtual screening in adenosine deaminase inhibitors detection and in-vitro study DARU J Pharmaceut Sci 2012 20 64 69 10.1186/2008-2231-20-64 PMC3556010 23351306

[B33] Noushini S Emami S Safavi M Kabudanian Ardestani S Gohari AR Shafiee A Foroumadi A Synthesis and cytotoxic properties of novel (*E*)-3-benzylidene-7-methoxychroman-4-one derivatives DARU J Pharmaceut Sci 2013 21 31 10.1186/2008-2231-21-31 PMC3668990 23587260

[B34] Aryapour H Riazi GH Ahmadian S Foroumadi A Mahdavi M Emami S Induction of apoptosis through tubulin inhibition in human cancer cells by new chromene-based chalcones Pharm Biol 2012 50 1551 1560 10.3109/13880209.2012.695799 22984888

[B35] Mosmann T Rapid colorimetric assay for cellular growth and survival: application to proliferation and cytotoxicity assays J Immunol Methods 1983 65 55 63 10.1016/0022-1759(83)90303-4 6606682

[B36] Nadri H Pirali-Hamedani M Moradi A Sakhteman A Vahidi A Sheibani V Asadipour A Hosseinzadeh N Abdollahi M Shafiee A Foroumadi A 5,6-Dimethoxybenzofuran-3-one derivatives: a novel series of dual Acetylcholinesterase/Butyrylcholinesterase inhibitors bearing benzyl pyridinium moiety DARU J Pharmaceut Sci 2013 21 15 23 10.1186/2008-2231-21-15 PMC3599263 23445881

